# Long‐Term Follow‐Up After Acute Gastroenteritis Caused by *Giardia* Infection in Juvenile Dogs

**DOI:** 10.1111/jvim.70123

**Published:** 2025-05-31

**Authors:** Karla C. Walz, Jan S. Suchodolski, Melanie Werner, Felix Grimm, Manuela Schnyder, Yury Zablotski, Stefan Unterer

**Affiliations:** ^1^ Clinic for Small Animal Medicine, Vetsuisse Faculty University of Zurich Zurich Switzerland; ^2^ Gastrointestinal Laboratory, Department of Small Animal Clinical Sciences Texas A&M University, TAMU Texas USA; ^3^ Institute of Parasitology, Vetsuisse Faculty University of Zurich Zurich Switzerland; ^4^ Clinic of Small Animal Medicine Centre for Clinical Veterinary Medicine, LMU Munich Munich Germany

**Keywords:** canine, enteropathy, gastrointestinal, *Giardia*, IBD

## Abstract

**Background:**

In humans, there is a high prevalence of postinfectious irritable bowel syndrome (IBS) after acute giardiasis.

**Objective:**

To evaluate the prevalence of chronic gastrointestinal and dermatologic signs in dogs after acute *Giardia*‐associated gastroenteritis.

**Animals:**

Forty‐nine dogs with acute gastroenteritis and confirmed *Giardia* infection and fifty control dogs without a history of acute giardiasis.

**Methods:**

Retrospective longitudinal study. Data were collected from dogs with acute gastrointestinal signs and confirmed *Giardia* infection at a young age (< 9 months) and from healthy controls matched by breed, sex, and age. After a minimum follow‐up period of 12 months, dog owners completed a questionnaire assessing chronic gastrointestinal and dermatologic signs later in life. Severity of chronic disease was quantified using a modified canine inflammatory bowel disease activity index (CIBDAI). Univariable logistic regression was used to compare frequencies of chronic signs between groups.

**Results:**

Dogs with acute giardiasis at a young age had a higher prevalence of chronic intestinal signs (*Giardia* 29%, 14/49; controls 10%, 5/50; *p* = 0.03) and pruritus (*Giardia* 33%, 16/49; controls 8%, 4/50; *p =* 0.01) later in life than did control dogs. A high canine acute diarrhea severity (CADS) index during acute enteritis, combined with metronidazole treatment, increased the risk of developing chronic gastrointestinal signs (*p* = 0.04).

**Conclusion and Clinical Importance:**

Juvenile dogs with acute gastroenteritis and confirmed *Giardia* infection had a higher prevalence of pruritus and chronic gastrointestinal signs. Severe enteritis and metronidazole administration may increase the risk of developing chronic gastrointestinal signs.

**Abbreviations**
AHDS: acute hemorrhagic diarrhea syndrome
CADS: Canine Acute Diarrhea Severity Index
CIBDAI: Canine IBD Activity Index
CPV: canine parvovirus
IBD: inflammatory bowel disease
IBS: irritable bowel syndrome

## 1 Introduction

In human medicine, acute enteritis is a potential risk factor for the development of chronic gastrointestinal and extraintestinal disease. The most important long‐term sequelae affecting the intestinal tract include irritable bowel syndrome (IBS) and inflammatory bowel disease (IBD) [1, 2]. The main extraintestinal consequences include reactive arthritis, Guillain–Barré syndrome, and hemolytic uremic syndrome [3, 4].

Data concerning the long‐term consequences of acute gastrointestinal disorders in dogs are sparse. Currently, three studies indicate that severe acute mucosal damage can lead to chronic gastrointestinal disorders. Compared with control dogs, dogs with a previous episode of acute enteritis caused by either canine parvovirus (CPV) infection or acute hemorrhagic diarrhea syndrome (AHDS) are more likely to develop chronic gastrointestinal signs later in life [5, 6, 7].

The mechanisms involved in the development of chronic gastrointestinal disorders after an acute insult are not yet fully understood. During the acute phase of severe intestinal disease, which is characterized by epithelial necrosis, the potential exists for permanent alterations in mucosal structures (e.g., villus blunting, destruction of the brush border, fibrosis). However, there is more evidence that in acute disease, barrier dysfunction as well as dysbiosis can lead to loss of oral tolerance and sensitize the immune system to food components and the intestinal microbiota. Owing to the destruction of the epithelial mucosal barrier (e.g., villous and mucosal necrosis in CPV enteritis and AHDS), an increased number of food antigens and bacteria can pass through the intestinal barrier and potentially influence the immune system [8, 9, 10]. Intestinal dysbiosis caused by antibiotic use during the acute phase of enteritis has been shown to be an additional risk factor with long‐term effects on metabolic and gastrointestinal health [2]. Disruptions in the composition of gut microbes, intestinal permeability, or both during early life, a period characterized by an immature immune system and an unstable unbalanced microbiome, pose an additional risk for the onset of chronic disorders. The gut microbiome in humans is particularly susceptible during the first three years of life, creating a critical window of opportunity in early development [11]. In dogs, major changes in the intestinal microbiome appear to occur in the first 6 months of life [12].

In human medicine, the risk for developing chronic disorders after acute infectious enteritis also depends on the causative agent [13]. The risk of developing postinfectious IBS is known to be higher in patients with enteritis caused by protozoa or other parasites. A total of 1252 people with verified giardiasis during an outbreak in 2004 in Bergen, Norway were evaluated after 3 years, and development of long‐term sequelae was assessed using a questionnaire. The prevalence of postinfectious IBS reported in this study was 47.9% (339/707) in the exposed group and only 14.3% (149/1042) in the control group [14].

*Giardia* is highly prevalent, especially among young dogs, and is thus commonly found in dogs with and without acute diarrhea. The severity of the disease depends primarily on the immune competence and age of the dog, with conditions ranging from mild to severe and, in some cases, life‐threatening [15]. The expert recommendation is to administer treatment (fenbendazole or metronidazole) to dogs displaying clinical signs [16]. It is unknown whether *Giardia* infections can lead to chronic gastrointestinal disorders in dogs. Nonetheless, acute enteritis can lead to intestinal barrier dysfunction, and metronidazole administration can lead to clinically relevant intestinal dysbiosis, two main factors suspected of contributing to chronicity.

Our aims were to evaluate the prevalence of chronic gastrointestinal and dermatologic signs in dogs after acute enteritis associated with *Giardia* infection as juveniles and identify possible risk factors triggering these chronic gastrointestinal signs and pruritus (young age and treatment with antimicrobials).

Our hypothesis was that there would be a high prevalence of chronic gastrointestinal and dermatologic signs after acute *Giardia* infection in young dogs.

## 2 Materials and Methods

### 2.1 Study Design

Our study was conducted as a retrospective longitudinal study. Data were collected from dogs that had acute gastrointestinal signs and confirmed *Giardia* infection at a young age (< 9 months) and from healthy controls matched by breed, sex, and age, for which follow‐up of at least 12 months was available. Dogs in the *Giardia* and control groups were identified by reviewing medical records from the Institute of Parasitology and the Clinic for Small Animal Internal Medicine of the Vetsuisse Faculty, University of Zurich, Switzerland. After giving consent, owners of dogs that met the criteria for participation in the study were provided with a questionnaire.

### 2.2 *Giardia* Group

The medical records of dogs with confirmed *Giardia* infection were retrospectively searched between January 2017 and December 2021. Diagnosis was made by fecal examination using the sodium acetate formalin concentration (SAFC) method, a coproantigen detection test using a commercial ELISA (RIDASCREEN, R‐biopharm, Germany), or by PCR detection of *Giardia* DNA [17, 18]. At the time of infection, the dogs had to be < 9 months old and have acute gastrointestinal signs (e.g., acute diarrhea, vomiting). A minimum follow‐up period of 1 year was required for all dogs. If the dogs were not treated at the Clinic for Small Animal Medicine, Vetsuisse Faculty, University of Zurich, the medical history of the primary veterinarian was requested. Dogs were excluded if they had a history of chronic gastrointestinal (e.g., chronic diarrhea, vomiting, weight loss), chronic skin disease (e.g., atopic dermatitis) or an acute intestinal disease (e.g., canine parvovirosis enteritis) associated with increased risk for chronicity before and at the time of *Giardia* infection.

### 2.3 Control Group

For the control group, we included dogs that were of the same breed, sex, and age (with a margin of ±1 year) as the dogs in the *Giardia* group and that were presented for regular health screenings, vaccinations, elective surgical procedures, or uncomplicated nongastrointestinal problems (e.g., bee stings) within a similar timeframe (±1 year). They were not included if they had a history of *Giardia* infection or signs of chronic gastrointestinal disease before the matched time of presentation. The exclusion criterion also included dogs undergoing treatment with immunosuppressive medications such as corticosteroids or antibiotics at the time of presentation. For a few dogs (e.g., rare breeds), no matched control dogs could be recruited.

### 2.4 Questionnaire

**FIGURE 1:**
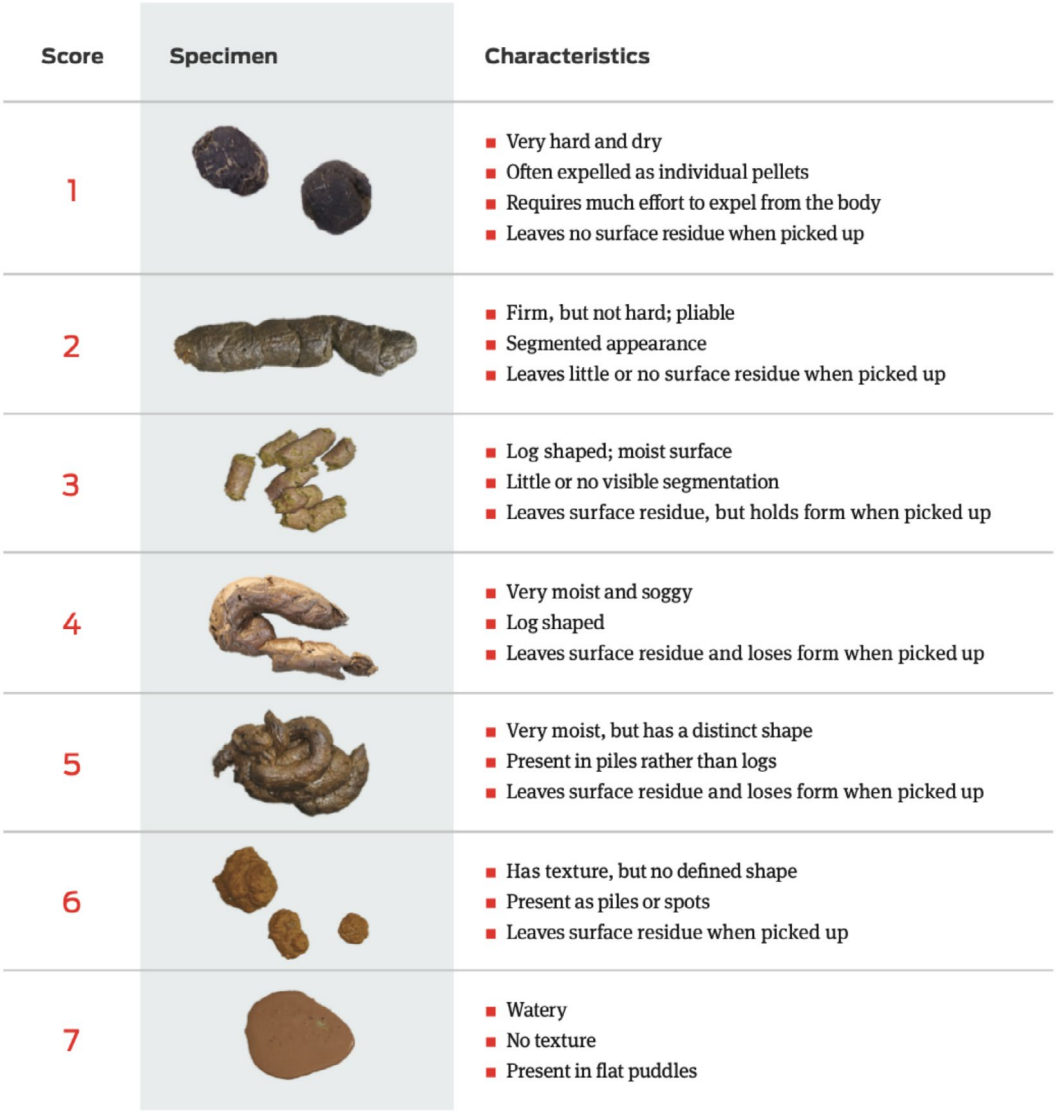
Purina fecal scoring chart; in a healthy dog, stool should ideally be scored 2.

The questionnaire was divided into three parts. The first part included general questions such as age of the dog, origin of the animal, diet, and health care routines (e.g., vaccinations, prophylaxis against endo‐ and ectoparasites). The second part focused on the period during which *Giardia* infection occurred, specifically inquiring about clinical signs, testing methods, and treatment protocols. The severity of the clinical signs was assessed using the Canine Acute Diarrhea Severity (CADS) index and the Purina fecal score (see Table 1; Figure 1). The third and final part concentrated on the health status of the animals from *Giardia* infection to the present day. Specific questions were asked about chronic gastrointestinal (GI) and dermatologic signs. The GI‐related questions focused on the period with the most severe clinical signs of chronicity. The severity of chronic gastrointestinal signs was quantified using a modified canine IBD activity index (CIBDAI; see Table 2) [19]. Chronicity was defined as episodes with signs of GI disease that lasted > 3 weeks or recurrent episodes of acute diarrhea that lasted > 7 days and occurred > 3 times per year. An owner observation period of at least 1 year was required to evaluate the development of chronic GI signs in both groups (*Giardia* and control). Similar questionnaires have been successfully used in previous studies [5, 7]. The questionnaire is provided as Supporting Information.

**TABLE 1:** Scoring system of the Canine Acute Diarrhea Severity (CADS) Index; severity levels: Insignificant (0–3); mild (4, 5); moderate (6–8); severe (≥ 9) disease.

Activity	0: Normal	1: Mild	2: Moderate	3: Severely decreased
Appetite	0: Normal	1: Mild	2: Moderate	3: Severely decreased
Vomiting	0: Normal	1: 1x/d	2: 2‑3x/d	3: > 3x/d
Fecal consistency	0: Normal	1: Moist, shaped	2: Pasty	3: Watery diarrhea
Frequency of defecation	0: Normal	1: 2‐3x/d	2: 4‑5x/d	3: > 5x/d

**TABLE 2:** Scoring system of the modified Canine IBD Activity Index (CIBDAI); severity levels: Insignificant (0–3); mild (4, 5); moderate (6–8); severe (≥ 9) disease.

Activity	0: Normal	1: Mild	2: Moderate	3: Severely decreased
Appetite	0: Normal	1: Mild	2: Moderate	3: Severely decreased
Vomiting	0: No Vomiting	1: 1x/w	2: 2‑3x/w	3: > 3x/w
Blood in stool	0: Normal	1: Mild	2: Moderate	3: Severe
Mucus in stool	0: Normal	1: Mild	2: Moderate	3: Severe
Fecal consistency	0: Firm, shaped	1: Moist, shaped	2: Pasty	3: Watery
Frequency of defecation	0: 1x/d	1: 2‐3x/d	2: 4‑5x/d	3: > 5x/d
Weight loss	0: No weight loss	1: Mild (< 5%)	2: Moderate (5%–10%)	3: Severe (> 10%)

### 2.5 Statistical Analysis

Chronic GI signs within the *Giardia* group (excluding the control group because of the absence of observations) were modeled as the response variable using multivariable logistic regression. Two backward variable selection procedures (one with interaction terms and one without) were performed using the glmulti R package. These analyses included four predictors: age at presentation, observation period, antibiotic administration, and disease severity (quantified using the CADS‐Index).

To evaluate differences in the frequency of chronic GI signs, pruritus, and dermatologic signs between the *Giardia* and control groups, univariable logistic regression was applied. Additionally, the Mann–Whitney *U* test was used to compare CIBDAI index scores between the groups.

Before conducting the primary statistical analyses, several variables were assessed using univariable analysis to ensure proper matching between the *Giardia* and control groups. These included age, observation period, sex, neuter status, breed, endo‐ and ectoparasite prevention, vaccination status, and weight. The Mann–Whitney *U* test was used to compare non‐normally distributed numerical or ordinal data, whereas normally distributed data were analyzed using a Student's *t*‐test. Categorical data were assessed using the Chi‐squared test. These univariable tests were used solely to confirm group comparability, with no further inferences drawn from them.

Throughout the study, a *p*‐value of < 0.05 was considered statistically significant.

## 3 Results

### 3.1 Demographics

For the final analysis, 99 completed questionnaires were available: 49 questionnaires from the *Giardia* group and 50 from the control group.

The *Giardia* group consisted of six mixed breed dogs (12%) and 43 pure breed dogs (88%), with the most common breeds being French Bulldog (*n* = 6), Labrador Retriever (*n* = 4), Flat‐Coated Retriever (*n* = 3), and Lagotto Romagnolo (*n* = 3). Twenty‐nine dogs were female (59%), and 20 were male (41%). At data evaluation, the median age was 4.9 years (range, 2.6–7.6), the median weight was 19.6 kg (range, 2.5–48), and the median time span of observation was 4.5 years (range, 2.4–7.2). Forty‐eight dogs were still alive at the time of data acquisition.

The control group included 10 mixed breed dogs (20%) and 40 pure breed dogs (80%). The most common breeds were French Bulldog (*n* = 6), Labrador Retriever (*n* = 5), Flat‐Coated Retriever (*n* = 3), and Lagotto Romagnolo (*n* = 3). Thirty‐seven dogs were female (74%), and 13 were male (26%), with a median age of 5.3 years (range, 2.7–8.5), a median weight of 20.0 kg (range, 3–45), and a median observation period of 3.6 years (range, 2.3–6.3). All dogs in the control group were alive at the time of data collection.

No significant differences were found between the *Giardia* group and the control group in terms of breed, sex, age, weight, prevention of endo‐ and ectoparasites, or vaccinations. However, the time span of observation was significantly shorter for the control group (*Giardia* median: 4.5 years; range, 2.4–7.2; control median: 3.6 years; range, 2.3–6.3; *p* ≤ 0.003; Table 3).

**TABLE 3:** Comparison of baseline data between *Giardia* and control group.

Variable	*Giardia* group	Control group	*p*
Breeds	88% Purebred (*n* = 43), French Bulldog (*n* = 6), Labrador Retriever (*n* = 4), Flat‐Coated Retriever (*n* = 3), Lagotto Romagnolo (*n* = 3), (other) 12% Mixed breed (*n* = 6)	80% Purebred (*n* = 40), French Bulldog (*n* = 6), Labrador Retriever (*n* = 5), Flat‐Coated Retriever (*n* = 3), Lagotto Romagnolo (*n* = 3), (other) 20% Mixed breed (*n* = 10)	0.29
Sex			0.12
Female	59% (*n* = 29)	74% (*n* = 37)	
Male	41% (*n* = 20)	26% (*n* = 13)	
Age (years)	Median 4.9 (range 2.6–7.6)	Median 5.3 (range 2.7–8.5)	0.26
Weight (kg)	Median 19.6 (range 2.5–48)	Median 20.0 (range 3–45)	0.88
Prevention of ectoparasites	Regularly 63% (*n* = 31)	Regularly 76% (*n* = 38)	0.17
	Not regularly 37% (*n* = 18)	Not regularly 24% (*n* = 12)	
Prevention of endoparasites	Regularly 76% (*n* = 37)	Regularly 76% (*n* = 38)	0.95
	Not regularly 24% (*n* = 12)	Not regularly 24% (*n* = 12)	
Vaccinations	Regularly 94% (*n* = 46)	Regularly 96% (*n* = 48)	0.63
	Not regularly 6% (*n* = 3)	Not regularly 4% (*n* = 2)	
Time span of observation (years)	Median 4.5 (range 2.4–7.2)	Median 3.6 (range 2.3–6.3)	> 0.003

### 3.2 Period During *Giardia* Infection

The median age at presentation with acute giardiasis was 4.6 months (range, 2–9). Twenty‐nine dogs (59%) were > 3 months at the time of presentation, and 20 dogs (41%) were ≤ 3 months old. All 49 dogs (100%) presented with acute diarrhea, and 16 dogs (33%) presented with acute vomiting. The median CADS index was 12.1 (range, 4–23), and the median Purina fecal score was 5.7 (range, 2.5–7). *Giardia* infection was diagnosed by SAFC in 33 patients (67%), ELISA in 15 patients (31%), and PCR in one patient (2%). Twenty‐three dogs (47%) received treatment exclusively with fenbendazole, whereas another 23 dogs (47%) were treated with metronidazole, either alone or in combination with fenbendazole. Treatment details were unavailable for three dogs (6%). Twenty‐one dogs (43%) underwent multiple treatments against *Giardia* during infection. Additionally, nine dogs (18%) experienced recurrent *Giardia* infections throughout their lives. Eight dogs (16%) had concurrent infections at the time of *Giardia* infection: three with *Isospora* spp., two with *Toxocara canis*, and one each with *Trichuris vulpis*, *Strongyloides stercoralis*, and *Coccidia*.

### 3.3 Period of Chronic Gastrointestinal Signs

**FIGURE 2:**
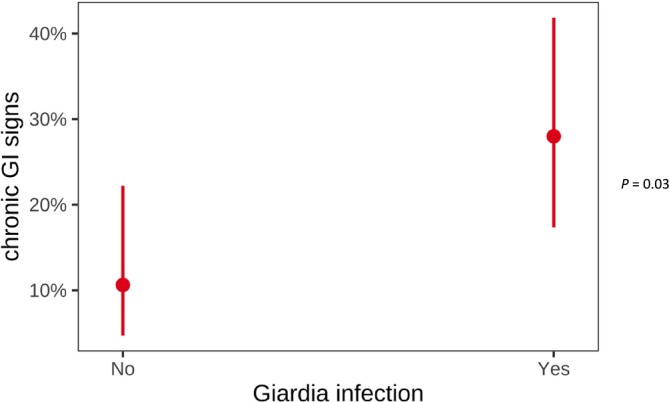
Comparison of chronic GI disease later in life between *Giardia* and control group.

Fourteen of 49 (29%) dog owners in the *Giardia* group and 5/50 (10%) in the control group reported chronic GI signs in their dogs later in life (Figure 2), indicating a higher prevalence of chronic GI disorders in young dogs with acute giardiasis (*p* = 0.03). Among the main clinical signs, chronic diarrhea was observed in all 14 dogs (100%) in the *Giardia* group, with 4 dogs (29%) also exhibiting chronic vomiting. In the control group, all 5 dogs (100%) experienced chronic diarrhea, whereas 1 dog (20%) also showed chronic vomiting. The clinical activity at the chronic phase in dogs with previous *Giardia* infection was scored as a median CIBDAI of 3 (range, 1–19), whereas the median CIBDAI score for control dogs was 1.6 (range, 0–6). Both groups had similar degrees of disease activity when they developed chronic GI signs (*p* = 0.32). Fecal consistency, assessed by the Purina fecal score, was similar: the median score for *Giardia* dogs was 2.9 (range, 1–7), and for control dogs was 2.4 (range, 2–6). In the *Giardia* group, most dogs (12/14) with chronic GI signs responded to dietary changes. The most frequently used diets were hypoallergenic (6 dogs), monoprotein (3 dogs) and highly digestible (3 dogs). One dog additionally required corticosteroids for resolution of chronic GI signs, and one dog received no treatment. In the control group, four dogs improved with dietary modifications, whereas one dog also required corticosteroids for resolution of chronic GI signs.

### 3.4 Period of Chronic Skin Signs

**FIGURE 3:**
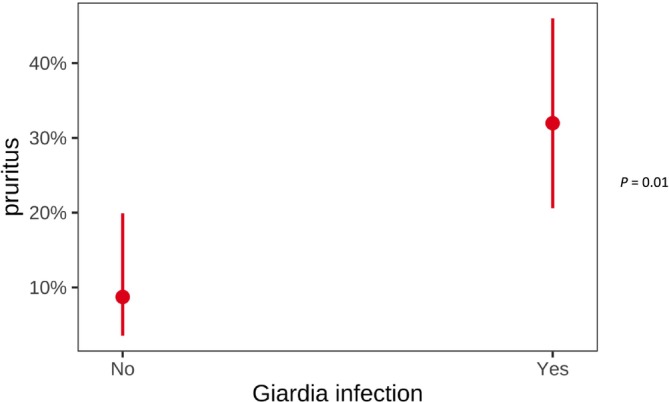
Comparison of pruritus later in life between *Giardia* and control group.

Sixteen of 49 (33%) dog owners in the *Giardia* group and 4/50 (8%) in the control group reported pruritus in their dogs later in life (Figure 3). Only 4/49 (8%) owners of dogs in the *Giardia* group and one in the control group mentioned a specific diagnosis of a skin disease. This finding indicates a higher frequency of pruritus in dogs that experienced acute giardiasis at a young age than in control dogs (*p* = 0.01).

### 3.5 Assessment of Risk Factors

**FIGURE 4:**
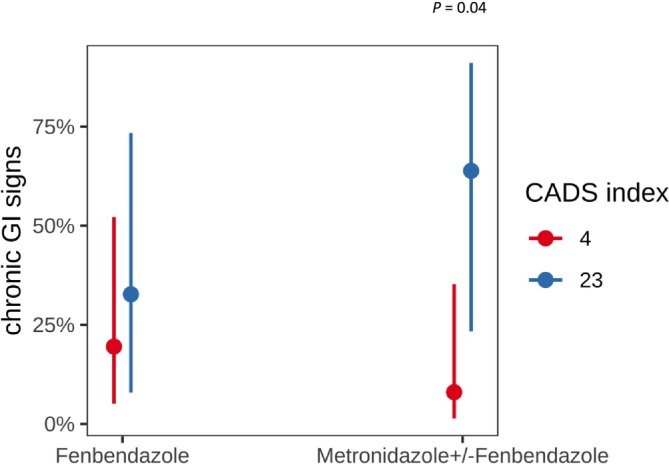
Prevalence of chronic GI signs in relation to treatment and disease severity.

When applying the backward selection approach without interaction terms, none of the predictors showed a significant association with the development of chronic GI signs, including antibiotic administration (*p* > 0.9) and disease severity, as measured by the CADS index (*p* = 0.13). However, when interactions between predictors were systematically analyzed using the glmulti algorithm, a significant interaction was identified (Figure 4). Specifically, a high CADS index (reflecting severe disease) during an episode of acute enteritis, in combination with metronidazole administration, was associated with a significantly increased likelihood of developing chronic GI signs compared with dogs with lower CADS scores (*p* = 0.04). In contrast, disease severity (CADS index) did not significantly affect the risk of chronic GI signs in dogs receiving only fenbendazole as an antiparasitic treatment (*p* = 0.59). This interaction emphasizes the important role of disease severity, in combination with antimicrobial treatment, in influencing the risk of chronicity.

## 4 Discussion

Our study indicated that dogs with *Giardia* infection as juveniles had a higher prevalence of chronic GI signs later in life than did dogs in the control group. This observation aligns with findings from previous studies involving dogs that experienced episodes of CPV and AHDS, which also showed increased risks for chronic GI diseases (CPV, 42%; AHDS, 28%) [5, 7]. The high prevalence of dogs with pruritus, along with chronic GI signs of moderate disease activity that improved with dietary changes, suggests that immune sensitization to food components may have developed in a subset of dogs during the acute phase of enteritis.

Dogs with hemorrhagic diarrhea caused by CPV or AHDS show destruction of the epithelial lining and are likely to have impaired intestinal barrier function. A recent study in dogs with AHDS demonstrated that a subpopulation with more severe clinical signs exhibited significant dysfunction of the intestinal barrier, as assessed by serum iohexol measurements [20]. An effective intestinal barrier facilitates the uptake of nutrients and fluids while concurrently preventing detrimental elements such as large‐sized food, antigens, toxins, and bacteria from traversing the intestinal epithelium into the underlying tissue [19]. There is evidence that in acute disease, barrier dysfunction can lead to loss of oral tolerance and sensitize the immune system to food components and the intestinal microbiota [21]. Owing to destruction of the epithelial mucosal barrier, an increased number of food antigens and bacteria can pass through the intestinal barrier and influence the immune system [22].

Currently, data on potential intestinal barrier dysfunction caused by *Giardia* infection are available from human medicine and from mouse models. In humans, *Giardia* infection leads to increased rates of enterocyte apoptosis and shortening of the brush border microvilli, with or without accompanying villus atrophy, resulting in intestinal barrier dysfunction [23]. This pathogenesis has been confirmed in mouse models and generally is hypothesized for different hosts, including dogs, for which the invasion of trophozoites leading to damaged brush borders has been confirmed [24, 25].

In our study, only dogs with signs of acute enteritis associated with *Giardia* infection were included. Although dogs with acute giardiasis do not normally exhibit hemorrhagic diarrhea, the initial contact or infection with *Giardia* in young dogs might result in disruption of the intestinal barrier. Additional studies are needed to assess the degree of intestinal permeability dysfunction during episodes of acute giardiasis and to evaluate the potential association between an altered intestinal barrier during acute enteritis and the development of chronic intestinal disease later in life.

One putative pathway for a protective effect against food allergen sensitization involves the intestinal microbiota [26]. This pathway was demonstrated in an experimental study in mice, where sensitization to a food allergen increased after antibiotic treatment [27]. Research in humans indicates that antibiotic‐induced dysbiosis might increase the risk of developing conditions such as asthma, allergic diseases, and IBD [28, 29, 30, 31].

In our study, no overall difference was observed in the development of chronic disorders between dogs treated with fenbendazole and those treated with metronidazole after experiencing enteritis associated with *Giardia* infection. However, our results suggest that the combination of severe enteritis and metronidazole treatment increases the risk of developing chronic GI signs. Both barrier dysfunction in severe enteritis and intestinal dysbiosis caused by antimicrobial treatment are considered potential mechanisms for triggering chronicity. It is likely that dogs with both factors have an even higher probability of developing chronic GI signs. Metronidazole can cause substantial, possibly long‐lasting, dysbiosis because of its anaerobic spectrum [32]. Thus, it should not be used as a first‐line drug in young dogs with *Giardia* infection. During infancy and early childhood, the immune system and the composition of the intestinal microbiome are not completely mature or stable. The intestinal microbiome follows a strict developmental maturation process as long as there is no substantial influence on the intestinal microbiota or interactions between the host and microbiome. In particular, delayed gut microbiota maturation during the first 100 days of life in humans can influence the development of allergic diseases and poor metabolic health [33]. In our study, no significant difference was found in the age at presentation among the *Giardia* group for dogs with and without chronic GI signs. However, in our study, we included only dogs < 9 months of age, and that had a median age of 4.6 months (range, 2–9 months) at the time of acute enteritis. Thus, we were not able to assess the difference between adult and young dogs, as well as between dogs > 2 months of age and very young puppies. Because the immune system seems to be vulnerable, especially during the first weeks of life, which represents a neonatal window of opportunity, very young puppies (< 2 months) might fail to develop a resilient intestinal microbiota after metronidazole‐induced dysbiosis and be at especially high risk of developing chronic intestinal disorders later in life.

Our study had several limitations. As a retrospective study based on owner‐reported questionnaires, it inherently is affected by variability and potential bias. Although the questionnaire provided valuable insights, the lack of standardized diagnostic evaluations limits the ability to establish a direct causal relationship between *Giardia* infection and chronic signs. To minimize misclassification, we attempted to obtain additional diagnostic information from private practitioners when possible.

The potential influence of other factors further complicates the interpretation of the results. A substantial proportion of dogs in the *Giardia* group required multiple treatments (43%) or experienced recurrent infections (18%), suggesting underlying conditions that could contribute to chronic signs. Additionally, 16% had concurrent parasitic infections, making it difficult to isolate the impact of *Giardia* infection.

Similarly, although pruritus was more common in the *Giardia* group, the underlying cause of pruritus could not be determined. Given that 37% of dogs in this group and 24% of controls lacked regular ectoparasitic prevention, flea infestation or flea allergy dermatitis could have contributed. Without dermatologic testing, we cannot confirm whether pruritus was directly related to *Giardia* infection.

Finally, the small sample size, particularly for dogs treated with metronidazole, limits our ability to fully assess the association between antimicrobial‐induced dysbiosis and chronicity. Additionally, although our data did not directly demonstrate a negative impact of metronidazole, its known potential to cause substantial, long‐lasting dysbiosis suggests that disease severity may play a key role in shaping treatment outcomes. Additionally, differences in the observation period between the *Giardia* and control groups may have influenced the results, and a longer observation period could allow for the detection of chronic conditions in more dogs.

In conclusion, our study contributes to the evidence that specific acute intestinal disorders potentially can lead to chronic disorders. Dogs with acute gastroenteritis and confirmed *Giardia* infection were found to have a higher prevalence of chronic GI signs and pruritus later in life. Our results also suggest that the combination of severe enteritis and metronidazole treatment may increase the risk of developing chronic GI signs. These findings must be interpreted in the context of the limitations of the study. Additional studies are needed to confirm our findings and identify key factors contributing to chronicity, such as young age, intestinal dysbiosis, barrier dysfunction, or type of infection. The knowledge gained might help establish new preventative treatment strategies.

## Disclosure

Authors declare no off‐label use of antimicrobials.

## Ethics Statement

Authors declare no institutional animal care and use committee or other approval was needed. Authors declare human ethics approval was not needed.

## Conflicts of Interest

The authors declare no conflicts of interest.

## Supporting information


**Data S1.** Supplementary Information.
