# Supplementary material for: Long‐Term Follow‐Up After Acute Gastroenteritis Caused by Giardia Infection in Juvenile Dogs
Source: J Vet Intern Med. 2025 May 31;39(4):e70123. doi: 10.1111/jvim.70123 (PMC12125909; doi:10.1111/jvim.70123)
Supplement: Supplementary file 1 — Data S1. Supplementary Information. [file JVIM-39-e70123-s001.pdf]

Questionnaire for Owners Whose Dog Has Suffered from Giardiosis

Note: The questionnaire is divided into three parts: Part A deals with general information about your dog. Part B focuses on the time BEFORE the Giardia infection, and Part C on your dog's health status AFTER the Giardia infection up to the present.

General Information

Your name: ____

Name of your dog: ____

Date of birth of your dog __ / __ / ____

Breed of your dog: ____

Gender of your dog: o female o male o neutered

Weight of your dog ___ kg

Veterinarian treating your dog:

____

Do you agree that we may contact your veterinarian for existing lab results/blood values/findings?

o yes o no

Part A: *General Background*

Does your dog live with you?	o yes	o no
Where did you get your dog (breeder/shelter/other)?		
Since when has your dog been in your possession?		
If not since puppyhood, where did your dog live before?		
Is your dog still alive<br><br>If not…<br><br>When did your dog pass away?<br><br>What was the cause of death/ reason for euthanasia?	o yes	o no
What do you feed your dog (dry food/wet food/self-cooked/raw food which brand)?<br><br>What did you feed your dog during its 1st year of life?		
Is your dog regularly vaccinated?<br><br>What has it been vaccinated against?<br><br>When was the last vaccination?	o yes	o no

Is your dog dewormed regularly?<br><br>How often is your dog dewormed?<br><br>When was the last deworming?<br><br>What product is your dog dewormed with?	o yes	o no
Is your dog regularly treated prophylactically against ectoparasites (fleas, ticks)?<br><br>How often is your dog treated against ectoparasites?<br><br>When was the last treatment?<br><br>Which product does your dog receive against ectoparasites?	o yes	o no
Does your dog suffer from a chronic illness?<br><br>If so...<br><br>What is it?<br><br>How long has the disease been known?	o yes	o no
Is your dog on long-term medication?<br><br>If yes...<br><br>What kind of medication is it?<br><br>How long has he been on them?	o yes	o no
Has your dog been on long-term medication in the past?<br><br>If yes...<br><br>What kind of medication was it?<br><br>When did he receive them?<br><br>How long did he receive them?	o yes	o no

Part B: *Health status* ***BEFORE*** *the Giardia infection*

Has Giardia been detected in your dog in the past?<br><br>If yes...<br><br>When was Giardia detected in your dog?<br><br>Did your dog show symptoms?<br><br>If yes...<br><br>What were the symptoms (diarrhoea, vomiting...)?<br><br>What was the main symptom?<br><br>Severity based on *CADS index*:	o yes<br><br><br><br><br><br><br><br>o yes	o no<br><br><br><br><br><br><br><br>o no

CADS-Index
Please tick as appropriate

How was your dog's activity?	o normal	o mild	o moderate	o severely decreased	o I cannot remember
How was your dog's appetite?	o normal	o mild	o moderate	o severely decreased	o I cannot remember
Did your dog vomit?	o normal	o 1x/day	o 2-3x/day	o >3x/day	o I cannot remember
Did your dog have blood in its faeces?	o no blood	o mild	o moderate	o severe	o I cannot remember
Did your dog have mucus in its faeces?	o no mucus	o mild	o moderate	o severe	o I cannot remember
How many times a day did your dog defecate?	o 1x/day	o 2-3x/day	o 4-5x/day	o >5x/day	o I cannot remember

What was the consistency of your dog's faeces?	o normal	o moist, shaped	o pasty	o watery diarrhea	o I cannot remember
Faeces quality based on *Purina fecal score*		What number would you assign to your dog's faeces?		__	

Score	Specimen	Characteristics
1	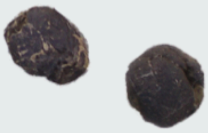	■ Very hard and dry<br>■ Often expelled as individual pellets<br>■ Requires much effort to expel from the body<br>■ Leaves no surface residue when picked up
2	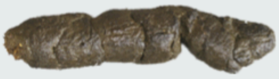	■ Firm, but not hard; pliable<br>■ Segmented appearance<br>■ Leaves little or no surface residue when picked up
3	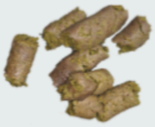	■ Log shaped; moist surface<br>■ Little or no visible segmentation<br>■ Leaves surface residue, but holds form when picked up
4	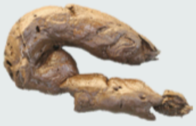	■ Very moist and soggy<br>■ Log shaped<br>■ Leaves surface residue and loses form when picked up
5	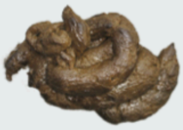	■ Very moist, but has a distinct shape<br>■ Present in piles rather than logs<br>■ Leaves surface residue and loses form when picked up
6	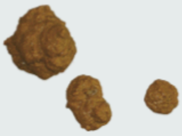	■ Has texture, but no defined shape<br>■ Present as piles or spots<br>■ Leaves surface residue when picked up
7	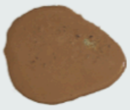	■ Watery<br>■ No texture<br>■ Present in flat puddles

What method was used to detect Giardia?<br><br>Which product was used to treat your dog for Giardia (antiparasitic fenbendazole (Panacur)/antibiotic metronidazole (Flagyl))?<br><br>How long was your dog treated?<br><br>How quickly did clinical improvement occur?<br><br>Was your dog treated more than once for Giardia during the infection?<br><br>If yes...<br><br>What medication was he treated with again?<br><br>Has your dog had other Giardia infections during its life?<br><br>When were these episodes and how often did they occur?<br><br>Were they treated?	o yes<br><br>o yes<br><br>o yes	o no<br><br>o no<br><br>o no
Was there another diagnosis at the time of the Giardia infection (e.g. parvovirosis)?<br><br>If yes...<br><br>Which one?	o yes	o no
Did your dog suffer from a chronic immunological disease before and at the time of the Giardia infection?<br><br>What disease was it?<br><br>What medication was used therapeutically?	o yes	o no

Did your dog suffer from a chronic gastrointestinal disease before the Giardia infection?<br><br>If yes...<br><br>After how long did the chronic symptoms disappear?	o yes	o no
Did your dog suffer from a skin disease before the Giardia infection?<br><br>If yes...<br><br>What type of skin disease was it?<br><br>What medication was used therapeutically?<br><br>Did your dog show itching before the Giardia infection?	o yes<br><br><br><br><br><br><br><br><br><br>o yes	o no<br><br><br><br><br><br><br><br><br><br>o no

Part C: *Health status **AFTER** the Giardia infection*

Does/did your dog suffer from a chronic gastrointestinal disease after the Giardia infection?<br><br>If yes...<br><br>At what age did the symptoms first appear?<br><br>After how long did the symptoms disappear?<br><br>What are the symptoms?<br><br>Severity based on *CIBDAI score*:<br>Refers to the period with the most severe clinical signs of the chronic disease	o yes	o no

CIBDAI-Score
Please tick as appropriate

How is your dog's activity?	o normal	o mild	o moderate	o severely decreased
How is your dog's appetite?	o normal	o mild	o moderate	o severely decreased
Does your dog vomit?	o no vomiting	o 1x/week	o 2-3x/week	o >3x/week
Does your dog have blood in its faeces?	o normal	o mild	o moderate	o severe
Does your dog have mucus in its faeces?	o normal	o mild	o moderate	o severe
How often does your dog defecate per day?	o 1x/day	o 2-3x/day	o 4-5x/day	o >5x/day
Has your dog lost weight?	o no weight loss	o mild (<5%)	o moderate (5-10%)	o severe (>10%)
What is the consistency of your dog's faeces?	o firm, shaped	o moist, shaped	o pasty	o watery
Faeces quality based on *Purina fecal score*<br><br>What number would you assign to your dog's faeces?	__			

Score	Specimen	Characteristics
1	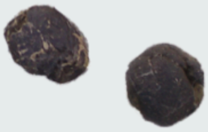	■ Very hard and dry<br>■ Often expelled as individual pellets<br>■ Requires much effort to expel from the body<br>■ Leaves no surface residue when picked up
2	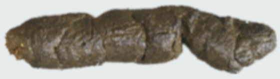	■ Firm, but not hard; pliable<br>■ Segmented appearance<br>■ Leaves little or no surface residue when picked up
3	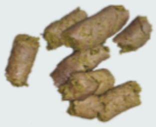	■ Log shaped; moist surface<br>■ Little or no visible segmentation<br>■ Leaves surface residue, but holds form when picked up
4	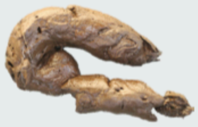	■ Very moist and soggy<br>■ Log shaped<br>■ Leaves surface residue and loses form when picked up
5	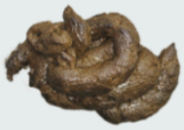	■ Very moist, but has a distinct shape<br>■ Present in piles rather than logs<br>■ Leaves surface residue and loses form when picked up
6	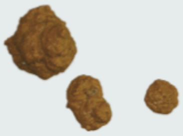	■ Has texture, but no defined shape<br>■ Present as piles or spots<br>■ Leaves surface residue when picked up
7	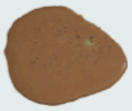	■ Watery<br>■ No texture<br>■ Present in flat puddles

What is your dog's primary gastrointestinal problem?		
How often does your dog suffer/ suffered from gastrointestinal problems since the Giardia infection?		
Average duration of symptoms?		
Frequency of the symptoms?		
Is your dog currently under therapy or has your dog been under therapy in the past?	o yes	o no
If yes...		
Did the symptoms improve/disappear as a result?	o yes	o no
What therapy is/was your dog receiving?		
Does/did your dog receive a special gastrointestinal diet?	o yes	o no
If yes...		
Which one (brand, home-cooked)?		
Did the symptoms improve/disappear?	o yes	o no
Did the chronic symptoms first appear after the Giardia treatment?	o yes	o no

Has your dog ever had an ultrasound or endoscopy?<br>*Please tick the appropriate box*<br><br>If yes...<br><br>What were the findings?	o ultrasound	o endoscopy

Does your dog show itching in addition to the gastrointestinal complaints?	o yes		o no
If yes...<br><br>How severe is the itching?<br>*Please tick as appropriate*	o Almost persistent itching. Itching does not stop when the dog is distracted, even itching in the treatment room.<br><br>o Prolonged episodes of itching occur when the dog is awake. Itching also occurs at night or wakes the dog up. Also occurs when the dog is playing, eating, exercising or otherwise occupied.<br><br>o Regular episodes of itching are observed when the dog is awake. Itching also occurs at night or wakes the dog up. No itching when the dog is playing, eating, exercising or otherwise occupied.<br><br>o Episodes of itching are often observed when the dog is awake. No itching when the dog is playing, eating, moving or otherwise occupied.<br><br>o Occasional episodes of itching, slight increase in itching since the onset of the disease.<br><br>o Symptom-free dog, no itching, itching has not increased since the onset of the disease.		
What came first?<br>*Please tick as appropriate*	o itching		o gastrointestinal complaints
Did the itching disappear/improve under glucocorticoid therapy (cortisone)?	o yes	o no	o never received

Does your dog suffer from a skin disease?	o yes	o no
If yes...		
What symptoms does your dog show?		
Which areas are affected?		
At what age did the symptoms first appear?		
Did the symptoms first appear after Giardia treatment?	o yes	o no
Do the symptoms occur seasonally?	o yes	o no
In which season are the symptoms worst?		
Has your dog ever had an allergy test (intradermal test or serum allergy test)?	o yes, both or only…<br>o intradermal test or<br>o serum allergy test	o no
What were the findings?		
Is your dog currently under therapy or has your dog been under therapy in the past?	o yes	o no
Did the symptoms improve/disappear as a result?	o yes	o no
What medication(s) is/was your dog receiving?		

Does your dog suffer from a food allergy?	o yes	o no
If yes...		
How long has this been known?		
Was it known before and at the time of the Giardia infection?	o yes	o no
Has your dog ever been fed an elimination diet or a hydrolysed/allergenic diet?	o yes	o no
If yes...		
When was your dog given the elimination diet?		
Did it lead to an improvement?	o yes	o no

Additional remarks

____

____

____

____

____

____

____
